# Heat-related illnesses: a scoping review of health system strategies, emergency responses and interventions in heat-prone areas

**DOI:** 10.1186/s12889-025-24012-4

**Published:** 2025-08-06

**Authors:** Peace U. Bassey, Faith A. Ngwu, Obinna C. Shimobi, Amarachukwu B. Isiaka, Chigbo C. God’swill, Christine Hosein, Benson C. Ephraim-Emmanuel

**Affiliations:** 1 School of Public Health, University of Port Harcourt, Port Harcourt, Rivers Nigeria; 2 Mechanical and Civil Engineering Contractors, Doha, Qatar; 3 London, United Kingdom; 4 College of Health Technology, Ogbia, Bayelsa Nigeria

**Keywords:** Extreme heat events, Heat-related illness, Health system resilience, Emergency response, Public health intervention strategies

## Abstract

**Background and aim:**

In order for effective heat emergency responses to be achievable, it is essential that healthcare delivery systems are made as heat-resilient as possible, alongside adoption of region-specific public health interventions. This review was thus aimed at identifying health system strategies, emergency responses and public health interventions adopted to ameliorate the harmful health effects of heat-related illnesses in heat-prone areas of the world.

**Methods:**

We performed a scoping review whose documentation was guided by the Preferred Reporting Items for Systematic Reviews and Meta-Analyses Extension for Scoping Reviews. Literature included were original articles published in English, policy review documents and program evaluations, population-based intervention studies, and other relevant gray literature. An electronic search of the PubMed, Web of Science, and World Health Organization (WHO) Global Health Observatory databases was performed in October 2024, using search terms developed from the search keywords.

**Results:**

Thirteen studies were included in this review, out of which 6 were policy-based documents on heat action plans while 7 were observational studies describing the application and effectiveness of heat action plans as well as susceptible members of populations to heat-related illnesses (HRIs). Nine of them were conducted in High-Income-Countries (HICs), while 4 were conducted in Low- and Middle-Income-Countries (LMICs). Eight of these articles (61.5%) were categorized as having good quality, while others were of fair quality. The different studies were identified to report various health system implementation strategies of heat action plans (HAPs), the targeted populations of these HAPs, emergency response actions, as well as public health interventions that contributed in reducing the impact of HRIs.

**Conclusions:**

Heat action plans are necessary to be tailored to the needs and resources of every heat-prone area, for effective development of heat-resilient health systems. Further research on quantifiable estimates of effects of HRIs prevention strategies are necessary to enhance their applicability during heat emergencies.

## Introduction

The human body temperature ranges between 36.5 and 37.5 °C which is achieved by balancing environmental heat exposures and metabolic processes mainly by evaporation [1]. However, with higher humidity, the body’s ability to cool itself reduces, and heat stress kicks-in, usually accompanied with the risk of adverse health outcomes [1–3]. The global problem of rising temperatures has been implicated in the increase of ambient temperature and humidity [1]. This potentially results in intolerable heat stress exposures [1], and the occurrence of a spectrum of heat-related morbidities and mortalities [1, 3–5]. These risk exposures are mostly worse during extreme heat events which have worsened over the years, and adversely affected the capacity of health systems and other critical infrastructure [4, 6]. 

Extreme heat events (EHEs), are periods of abnormally high ambient environmental temperatures [3, 7]. These EHEs occur as a result of the rising greenhouse gas emissions and significant use of fossil fuels, which are projected to raise global temperatures by up to 4 °C by 2110 [1]. Various intensities of EHEs have been reported around the world in the last two decades, and these have been reported to evoke global public health concerns [3]. Excessive heat loads impair the body’s ability to remove the excess heat, which also increases the body’s core temperature and metabolic activity, and result in a spectrum of heat-related illnesses [6]. This mostly occurs when the affected individual has exhausted relevant physiological compensatory mechanisms and is unable to carry-out behavioral steps to cool down [6]. This illness spectrum includes mild disease such as heat cramps and oedema, as well as moderate disease such as heat syncope and heat exhaustion due to heat-related vasodilation and dehydration. Symptoms of moderate forms of the disease however abate with rest, cooling, and rehydration [6]. These notwithstanding, when not promptly identified and treated, heat exhaustion (the commonest heat-related illness) can progress to heat stroke which is severe and life-threatening [3, 4]. The condition is characterized by hyperthermia, with a > 40 °C core temperature, multi-organ failure and neurological impairment, such as confusion, seizure, or coma [6]. Extreme heat-related illnesses can also exacerbate cardiovascular, respiratory, and renal diseases [3, 4]. In 2018, as much as 220 million cases of heatwave exposures which heavily impacted elderly persons, was reported [8, 9]. These EHEs have been reported in India, Central Europe, Western United States, Pakistan, China, Turkey and other locations around the world which show the global impact of these events [4, 7, 10]. Between the years 2000 and 2010 in the United States, over 30,000 hospitalizations for heat-related diseases were reported, which predominantly affected the elderly, athletes, and outdoor workers. As high as 75% of heat-related emergency department visits during that period, were also mostly due to heat exhaustion [11, 12]. 

Treatment modalities for someone experiencing heat-related illnesses include placing the patient in a supine position in a cool environment, cooling the head with cold water, moisturizing the skin, and placing of iced bags [1, 8]. Water and electrolytes also need to be replaced either orally or intravenously with continuous monitoring of vital signs [1, 8]. Cooling requires creating a gradient for heat loss from the skin to the environment by conduction, convection and/or evaporation [1, 13]. In pre-hospital scenarios, it is necessary to follow the rule “*cool first and transfer later*”, and continue cooling during transport with available means [1, 8]. Even though unmitigated EHEs are a risk to all persons in a population, some population groups are at higher risk due to individualized, behavioral, physiological, or disproportionate exposure-related factors that can limit the adaptation to hot and humid conditions [7, 10, 14]. These typically include elderly persons particularly those with multiple medical comorbidities [4, 14], pregnant women, homeless persons and those living in poor residential structures. Others include children and infants, poorly educated persons [3, 7, 10], alcohol and drug users, persons who work outdoors, those who engage in excessive exercise, among others [3, 4, 8]. 

The need for effective health systems to respond to heat extremes has become an urgent priority for health policy and practice, considering the current and projected worldwide increase in the frequency and intensity of EHEs [15]. Health systems need to become more heat resilient, in order to be able to reduce accompanying health care–related climate impacts of these EHEs [7]. In doing this, there is the need for preparedness for emergencies as this is essential for the preparedness of health organizations and systems for EHEs emergencies and disasters [9]. In order for health systems to become climate-resilient, each building block of the health systems at the local to national levels also need to be climate-resilient [16]. These building blocks include “*leadership and governance*,* available health workforce*,* health information systems*,* essential medical products and technologies*,* service delivery*,* and financing”.* These should be enhanced to effectively tackle healthcare emergencies related with worsening climatic conditions [3]. Preparedness activities commonly take the form of heat action plans (HAPs) which are policy documents that lay-out strategies to tackle EHEs, in order to reduce morbidity and mortality from these events [9]. These activities involve heat risk assessments, identifying and addressing heat-related problems, enhancing manpower capacities for effective response, ensuring adequate funding, and executing appropriate heat-mitigation actions [3, 9]. 

Despite the global prevalence of heat illness, no aggregation of reports stating the various health system strategies, emergency responses and public health interventions geared towards ameliorating the harmful health effects of heat-related illnesses in heat-prone areas has been done. Conducting this scoping review was thus beneficial as it has helped to aggregate this data that can be applied for developing strategic response plans to EHEs. This review was thus focused on identifying health system strategies, emergency responses and interventions to ameliorate the harmful health effects of heat-related illnesses in heat-prone areas of the world. Objectives of the review included:

- To outline current health system strategies and policies to address heat-related emergencies in these areas.
- To identify the targeted populations of these strategies and policies in heat-prone areas.
- To describe emergency response protocols and interventions to mitigate the harmful health effects of heat-related emergencies.
- To explore public health interventions designed to reduce the impact of heat-related illnesses.

## Materials and methods

The documentation of this scoping review was guided by the Preferred Reporting Items for Systematic Reviews and Meta-Analyses Extension for Scoping Reviews [17]. This type of review was done to to synthesise current literature necessary to answer the review questions raised in each of the above-stated objectives. It was also done to identify existing knowledge and gaps regarding the study objectives, with the view of providing a basis for further research.

### Study eligibility

In this scoping review, we focused on identifying health system strategies, emergency responses and evidence-based interventions to ameliorate the harmful health effects of heat-related illnesses in heat-prone areas of the world. Studies were deemed eligible if they extensively described health system strategies to tackle heat-related illnesses, and emergency responses to mitigate the harmful effects of heat-related illnesses. Studies showcasing evidence-based public health interventions to reduce the impact of heat-related illnesses on a population level were also seen as eligible, and there were no geographic constraints in this review. Other criteria used for inclusion eligibility of literature included the following:

- Original articles published in English or having complete English translations,
- Policy review documents and program evaluations written in English,
- Studies employing prospective, cross-sectional, or observational research methods centred around heat-related illness. In addition, surveillance studies. reports, as well as studies that described the epidemiologic profile of heat-related illness in different countries were included.
- Population-based intervention studies.
- Literature reviews and other relevant gray literature.
- Studies that employed qualitative research methods.

The following however characterized studies excluded from the review:

- Protocols, opinion pieces, and case reports.
- Studies assessing the impact of cold temperature on human health.
- Ex vivo/in-vitro/animal models’ studies.

### Literature search strategy

An electronic search of the PubMed, Web of Science, and World Health Organization (WHO) Global Health Observatory databases was performed in October 2024, and specific search terms used in each database are shown in Appendix 1. Citations and reference lists of studies that were obtained during the databases’ search were also cross-checked for identification of gray literature that pertained to the review objectives. The literature search strategy was mainly developed for advanced searching of the afore-mentioned databases. The search vocabulary and syntax were also adjusted across databases as shown in Appendix 1. The search was carried out using the ‘OR’ Boolean operator for single groups of synonymous keywords, and each single group was then combined using the ‘AND’ Boolean operator to produce a list of citations in the various databases. In addition, search limits included works published in English, and in peer-reviewed full-text articles. There was however no restriction on the duration of studies to be included in this study, to allow for identification of all studies published from inception till October 2024. Doing all these, ensured an optimal identification and location of relevant literature sources for this review. Search terms (See Table 1) were generated using the modified Population, Context and Outcome (PCO) framework [18]. In order to ensure a thorough search, a final search of databases was conducted to ensure that all relevant literature had been captured. The search for literature was considered to be complete when the same articles began to appear again in the electronic databases’ search.

**Table 1:** PCO elements used to articulate the search strategy

Criteria	Keywords used	Description
Population	“Persons”	Individuals residing in heat-prone areas
Context 1	“Heat-related illnesses”, “Heat-related problems”, “Heatstroke”, “Heat exhaustion”, “Heat syncope”	Manifestations of health problems associated with extreme-heat events.
Context 2	“Health system strategies”, health system policies	Plans, policies and strategies for management of heat-related illnesses.
Context 3	“Emergency preparedness”, “emergency response”	Emergency responses put in place to mitigate the health effects of the extreme-heat events
Outcome	“Public Health Interventions”	Available scores or rates of effect of public health interventions to mitigate health effects of heat-related illnesses

### Screening of literature

**Fig. 1:**
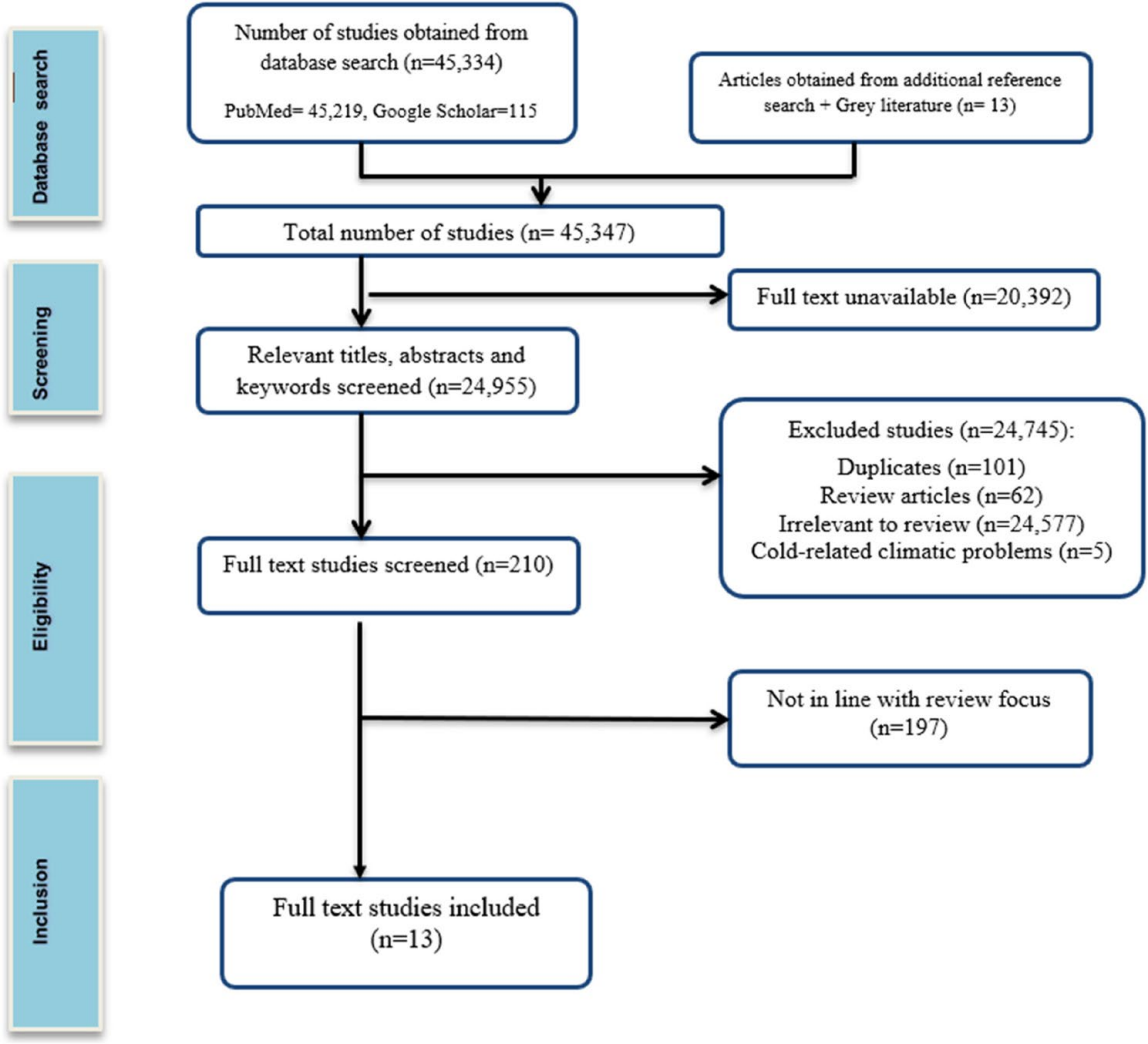
PRISMA flowchart for studies’ selection

Titles of all obtained literature were screened and their abstracts were read to identify relevant studies for further full-text screening and selection of all full-texts that met the eligibility criteria. All selected full-texts were then read, and suitability for inclusion was assessed. A Preferred Reporting Items for Systematic Reviews and Meta-Analyses (PRISMA) flowchart (See Fig. 1) [19], summarises this screening process.

### Data extraction and synthesis

Data items extracted included the author, year of publication, country, study characteristics (study design, population, sample size), and the findings from the study (in accordance with the review objectives). The primary outcomes of interest included information on evidence-based health system plans, policies and strategies; the targeted populations of these policies and strategies, emergency responses and public health interventions to mitigate the health effects of heat-related illnesses. Other outcomes of interest included the effectiveness of interventions, impact on morbidity and mortality rates, as well as barriers to effective preparedness or response, and areas for improvement.

As a result of the wide variation of studies in relation to study designs utilized, populations assessed, interventions and associated outcomes, it was decided that a narrative synthesis was the best approach for synthesising the findings of the different studies/literature evidence included in the review.

### Quality appraisal

Assessment using the studies’ methodologies revealed that articles included in the review ranged from policy documents, to time-series modelling and observational studies. The study quality assessment tools developed by the Joanna Briggs Institute [JBI] (to appraise the quality of textual evidence) [20], and the National Heart, Lung and Blood Institute [NHLBI] (to appraise the quality of observational research papers) [21], were thus used to assess the quality of included literature.

The JBI Tool had 7 questions with items centred around the stakeholders involved in developing the policy, report of methodologies used, congruence with extant literature among others. All questions had four responses, “Yes”, “No”, “Unclear” and “Not applicable”. Literatures were categorized as “Good” if 70% of all applicable responses were “Yes”, “Fair” if 40–70% of all applicable responses were “Yes”, and “Poor” if less than 30% of all applicable responses were answered “Yes”. The NHLBI as well had a range of 12 to 14 questions with items around clear objectives, defined population, sample size justification, clear definitions of exposure and outcome variables, among others. In this tool, all questions had three responses, “Yes”, “No” and “Not applicable”. The literature materials were categorized as “Good” if 80% of all applicable responses were “Yes”, “Fair” if 40–80% of all applicable responses were “Yes”, and “Poor” if less than 40% of all applicable responses were answered “Yes”. Two reviewers independently appraised randomly assigned articles and the occurrence of any mismatch was resolved via collaborative team discussions.

## Results

### Study selection

Altogether, 45,347 records were initially identified throughout all the perused databases, out of which 24,955 were identified to be free full text literature. Next, 101 duplicates were excluded and 24,854 had their titles and abstracts screened. The results were thus further narrowed down to 210 after screening the titles and abstracts of the literature for eligibility for this review, and literature were excluded because they were irrelevant to the review, were review-based studies, and dealt with cold-related climatic problems. Conducting a review of the 210 full-text articles resulted in the final inclusion of 13 full-text articles for use in this scoping review, after removal of studies not in line with the review focus (see Fig. 1).

### Study characteristics

Concerning the characteristics of the 13 studies included in this review, 6 were policy-based documents and reports on heat action plans while 7 were observational studies describing the application and effectiveness of heat action plans, as well as susceptible populations. Also, 9 of them were conducted in High-Income-Countries (HICs), while 4 were conducted in Low- and Middle-Income-Countries (LMICs).

Results from the quality appraisal of the articles used for this scoping review showed that 8 of them (61.5%) were categorized to be of good quality, while the others were graded as being of fair quality as shown in Table 2. All the observational studies provided clearly defined objectives, a defined population frame which mostly participated (where applicable), with well-defined exposure and outcome measures. Only some studies however justified their sample size and selection criteria. The study that applied the time series analyses was identified to properly define the underlying data collection and measurement techniques. In addition to these, the policy review documents were produced by all required stakeholders who had the necessary expertise to prepare them, methodologies used were well reported, and congruence with extant literature, where necessary, was made. Considering that the data analytical and documentation methodologies were strong and properly defined, the findings from the various included literature were considered reliable.

The different studies were identified to report various health system implementation strategies of heat action plans (HAPs), populations targeted by these HAPs, emergency response actions that were implemented, as well as public health interventions that contributed in the reduction of the impact of the heat-related illnesses. These can be seen in Table 2 and are further described in this section.

### Health system strategies/policies to address heat-related emergencies (Heat action Plans)

Most of the literature included in this review presented country-specific health system policies in the form of heat action plans that addressed problems of heat-related emergencies in the affected countries. This was done in both high and low/middle income countries, and altogether, 11 of the included literature provided information on these heat action plans. The National Action Plan for Climate Change and Human Health (NAPCCHH) is one of such action plans that was formulated in India with the vision to strengthen the healthcare services for citizens of India against the adverse impacts of climate change on health especially among vulnerable members of the population including children, women among others. It also had the goal to reduce morbidity, mortality, injuries and health vulnerability experienced by persons due to climate variability and extreme weathers. Key areas of this action plan involve tackling heat related illnesses, the creation of green and climate resilient healthcare facilities, as well as dealing with air pollution. The policy was centred around ensuring that health was adequately represented in the climate change agenda of the country, while strengthening health systems at all levels [22]. Another health system developing strategy that was identified to tackle the effects of heat-related events was the health early warnings system surveillance (HEWS) in Saudi Arabia. This system contributed in improving the timeliness of reporting of heat-related events and helped in detecting 2 events (heat-related illnesses and injuries/trauma) during the 2019 Hajj. This system used syndromic surveillance and event-based surveillance data, to generate automated alarms for public health events, which triggered alerts for rapid epidemiological investigations and facilitated the monitoring of health events [23]. 

In Canada, urban design models were also set-up to identify its capability in tackling HRIs. It involved landscape modifications that were designed to cool the environment and see its effects on the expected number of emergency medical-related calls in the neighbourhoods during extreme heat events. The cooling design strategies were identified to result in reduced energy overload on people, and resulted in the reduction in heat-related ambulance calls by up to 40–50%. In the redesigning efforts, reducing radiation exposure (short-wave solar and long-wave terrestrial radiation) was prioritized [24]. The Ahmedabad Municipal Corporation in India also set-up the Ahmedabad’s Heat Action Plan to serve as an early warning system and heat preparedness plan. The plan was instrumental in building the awareness of the public concerning the risks of extreme heat, as well as involved the training of medical and community workers to effectively prevent and respond to heat-related illnesses. This plan also developed a strategy to coordinate inter-agency emergency response in the event of any heat-related climatic event [25]. In Canada, another heat action plan was developed on the Island of Montreal, known as the “Montreal heat response plan”, which was also upgraded after the 2010 heat wave crisis in that area. It was designed to ensure the surveillance of weather and health indicators during the summer season for any heat-related health problem. It provided a way for coordinated actions to be carried to reduce the occurrence of morbidity and mortality secondary to heat effects, particularly when weather thresholds were reached or when the health indicators reflected a course for concern. The main reason for the upgrades at that time was the need to include initiatives to improve the communication of preventive measures and enhance early interventions during a heat wave [26]. 

In New Hampshire, United Kingdom, the excessive heat emergency response plan was also put in place to provide a threshold for heat response activation, a clear description of heat indices, associated health risks, response activities, as well as the criteria for setting-up cooling centres [27]. In the United States, there was also the provision of the extreme heat public health preparedness plans and response activities at the county level, after the occurrence of a summer of record-setting heat [28]. The heat alert response system is another health system strategy put in place by Health Canada to address emergencies related to HRIs in Canada. The heat response toolkit was intended for use by public health and emergency management officials involved in the development or update of heat-health communication strategies. Its design was primarily to guide the development of targeted heat-health communication campaigns to effectively educate the public, and provide necessary outreach products for specific audiences [29]. In Bangladesh, a set of national guidelines on heat-related illnesses were released. These basically focused on strengthening the healthcare system for heat-related events preparedness, ensuring sustainability of the healthcare delivery system, reducing the risk of HRIs, heat-related communication and community engagement and promoting intersectoral collaboration for effective action. Part of these guidelines were hinged on the premise that risk communication, community engagement, and awareness-raising campaigns were central to effective prevention and management of heat-related illnesses in the general population [1]. 

The Indian Institute of Public Health has also provided instrumental health system approaches targeting the mitigation of emergencies related with HRIs in Odisha. Strategies adopted included setting-up early warning protocols and ensuring system preparedness to cope with heatwave events, as well as providing and evaluating town-specific heat action plans, and ensuring that health advisories for enhancing the prevention the impacts of heat stress. This guide also acted as a guiding document for capacity development and decision-making of policymakers and healthcare managers regarding heat-health system preparedness and response at the state, district and sub-district levels [10]. Finally, in Europe, the World Health Organisation regional office released a heat action plan guidance document for implementation at national, regional and city-level domains. It provided information on heat- health warning systems, risk communication modalities for educating the public, as well as plans to ensure health- and social-system readiness to deal with heat-related events and health risks. The heat action plan also provided urban design plans to reduce these heat-health risks in the long-term, as well as surveillance, monitoring and evaluation of the heat-health action plans for monitoring health impacts and evaluating response measures during and after heat-wave events [15]. 

### Targeted populations of the HAPs

Out of the 13-literature evidence included in this scoping review, most of them described members of the population who were particularly targeted by the identified heat action plans (HAPs). In the literature evidence obtained from LMICs, it was identified that various population groups were mostly targeted in the HAPs. These included the elderly (60 years and above) [2], (65 years and above) [22], infants, children and adolescents, pregnant women, the mentally ill, chronically ill persons on medications, the homeless and poor, as well as the socially isolated. Others included construction workers, traffic police, as well as low-income persons [2, 10, 22, 25]. Among evidence obtained from HICs, targeted members of the population included those aged approximately 65–70 years of age [15, 26, 27, 29], those suffering from cardiovascular disease [15, 26], mental illness as well as chronically ill persons [26]. 

### Emergency response protocols/interventions to mitigate effect of heat-related emergencies

Out of thirteen studies included for this review, ten of them provided information on emergency response interventions used during heat-related emergencies to mitigate its effects on human health. In the health early warnings system surveillance (HEWS) carried out in Saudi Arabia during the 2019 Hajj, emergency response protocols included providing hospitals with the necessary equipment and trained personnel needed during that period in order to ensure that heat emergencies were properly managed. Other interventions included the provision of cooling units, as well as the release of guidelines necessary for pre- and in-hospital management of HRI cases during the Hajj season [23]. In another action plan (the Ahmedabad’s Heat Action Plan that served as an early warning system and heat preparedness plan), other emergency response protocols were used. These included the dissemination of regular heat alerts and information to local agencies and stakeholders using emails and text messages, as well as improving preparedness, information-sharing and coordination of heat wave response plans. Other response protocols include the continuous monitoring of heat illnesses and daily mortalities [25]. The Montreal heat response plan was identified to utilize local health departments to identify and protect vulnerable individuals, opening of air-conditioned shelters, as well as monitoring of the health surveillance screen used to provide real-time data of the heatwave situation [26]. 

In the United States of America, chemical cold packs, air-conditioning, the use of wet towels on affected person’s body, as well as the use of fluids for oral rehydration, served as emergency response interventions during heat-related events [30] Also in the United Kingdom, response teams were kept on high alert for mobilization, while medical supplies were deployed. In addition, cooling centres were opened, and there was the provision of orientation on guidelines for managing heat-related illnesses [27]. Other identified heat response emergency protocols included the provision of extended shelters for the homeless, active surveillance of HRI cases, the development of vulnerability heat maps [5], the use of chemical cold packs, air-conditioning, use of wet towels and oral rehydration with fluids [29]. The United Nations Children’s Fund (UNICEF) in Bangladesh in collaboration with the Bangladesh Government also ensured the readiness of response services and supplies for deployment during heat emergencies, advocated the wearing of loose clothing, and providing orientation to the public on guidelines for managing heat-related illnesses [2]. It is also essential that during heat-related events, heatwave hotspots and vulnerable members of the population are identified alongside activating heat alerts and Heat Action Plans to address heat-health risks as was done in India [10]. These were also carried out by the World Health Organisation Europe regional office who also ensured that cool areas were provided and maintained at temperatures below 26°c and disseminating necessary information to all concerned stakeholders on management protocols [15]. 

### Public health interventions to reduce the impact of heat-related emergencies

The provision of interventions to effectively reduce the exposure of the public to the impacts of heat-related illnesses is an important consideration in heat-prone areas. In this review, twelve of the included literature provided descriptions of public health interventions that were put in place to mitigate heat-related impacts on the public. In India, interventions utilized included strengthening heat and human health surveillance systems, building the capacity of the public to handle these heat-related events through heat-health awareness campaigns and community outreaches. Others included the standardization of the investigation process of deaths due to HRIs, developing and disseminating early warning systems and response measures, as well as developing state, district and city-specific heat action plans [22]. In Saudi Arabia during the 2019 Hajj, free health services were provided in hospitals and primary health centres, where daily and total consultations peaked during the period [23], and in the United States, public education and increase in access to electric fans/ air-conditioning were ensured [5] Access to electric fans/air-conditioning was also utilized in India and Australia [10, 28], while public health education on preventive measures, including the need to stay hydrated, wear light clothes, taking frequent showers/baths among others was utilized in other regions [2, 10, 15, 25–27]. 

In Canada, interventions put in place included the placement of trees to intercept direct solar radiation away from humans, buildings, parking lots; the use of solar radiation interceptive building materials (green roofs or high-α roofing material, as well as the use of light-colored (or higher-α) paving materials [24, 29]. This was also a public health measure to reduce the occurrence of HRIs in Bangladesh [2]. In Canada, pool opening hours were also extended, surveillance was intensified, and preventive measures were also applied by health care facilities [26]. In addition, targeted groups were provided with timely and accurate information on hydration, the use of cooling centres, and ensuring community designs that enabled easy access to heat-relief places and drinking water [29]. 

In Australia, the provision of cool drinking water was ensured, wearing broad brimmed hats and having shady rest areas were advocated, and work periods were also rescheduled to prevent unwarranted exposure during heat events [28] In India, digital visual displays (showing prevailing temperatures, information of heat illness symptoms and precautions) were also put into use, all city gardens were kept open during afternoon hours to provide shaded areas, and all medical and paramedical staff received reorientation on managing HRIs [10, 25]. In Bangladesh and certain parts of Europe, there was also the use of hollow blocks for buildings and retrofitting of some building envelopes and insulation respectively. Both regions also used solar reflective paint, double-glazed windows, roof-cooling materials, and building designs that ensured cross-ventilation, as effective interventions to limit the impact of heat events [2, 15]. In Europe, other measures advocated for use included ensuring efficient active cooling, and the provision of external shading [15]. 

**Table 2:** Characteristics of included studies

Author/ (Year)	Country	Study design/ Study population/ Sample size		Health system strategies to address emergencies due to HRIs	Members of the population targeted by these strategies	Emergency response protocols to mitigate harmful effects of HRIs	Public health interventions designed to reduce the impact of heat-related illnesses	Quality
1. Kumar et al. 2020 [22]	India	Policy Document/ General Indian population		National Action Plan for Climate Change and Human Health (NAPCCHH)	People aged 65 and older, infants and young children, pregnant women, people with chronic medical conditions, people on medications, outdoor workers, destitute and low-income population	-	- Strengthening the Heat and Human Health Surveillance System and Capacity Building. - Standardizing the Investigation of Deaths due to HRIs. - Developing State/ District/ City Specific Heat and Health Action Plan. - Increasing Public Awareness and Community Outreach. - Developing Measures for Early Warning System/ Alerts and Response.	Good
2. Bieh et al. 2020 [23]	Saudi Arabia	Observational study/ Emergency and outpatient departments of 16 Ministry of Health hospitals		Health Early warnings system surveillance (HEWS)	-	- Providing hospitals with the necessary equipment and trained personnel. - Provision of cooling units. - Provision of guidelines for pre- and in-hospital management of HRI cases during the Hajj season	- Free health services during the Hajj rituals in hospitals and primary health centres, which saw the daily and total consultations peaking during that period.	Fair
3. Graham et al. 2017 [24]	Canada	Time series modeling/ City of Toronto		Urban design as a heat response strategy	-	-	-Placement of trees to intercept direct solar radiation away from humans, building, parking lots. - Provision of trees to provide shade. - Use of solar radiation interceptive building materials (green roofs or high-α roofing material. - Use of light-colored (or higher-α) paving material	Good
4. Xiang et al. 2016 [28]	Australia	Cross-sectional/ Outdoor industry workers/ 749		-	-	-	- Provision of cool drinking water. - Wearing broad brimmed hats - Rescheduling work time - Central cooling and air conditioning. - Having a shady rest area	Fair
5. Shah et al. 2014 25]	India	Policy/Program document / Residents of Ahmedabad Municipal Corporation		Ahmedabad’s Heat Action Plan	- Children and other vulnerable members of the population	- Dissemination of regular heat alerts and information to local agencies and stakeholders through emails and text messages. - Improved preparedness, information-sharing and coordination of heat wave responses - Continuous monitoring of heat illnesses and daily deaths	Use of digital visual displays (containing the temperature, information of heat illness symptoms and precautions). - All city gardens kept open during afternoon hours to provide shaded areas. - Procuring weather gauge instruments for better local monitoring of temperature - Media engagements - Reorientation of all medical and paramedical staff	Good
6. Price et al. 2013 [26]	Canada	Surveillance program evaluation/ All residents of the Island of Montreal		Montreal Heat Response Plan	- Individuals over approximately 70 years of age and suffering from cardiovascular disease. - Persons with mental illness	- Involvement of local health departments to identify vulnerable individuals. - Opening of air-conditioned shelters. - Monitoring of the health surveillance screen	- Mass media communication and education campaigns. - Extension of pool opening hours. - Advisories to the public via different media on preventive measures. - Intensification of surveillance and application of preventive measures by health care facilities.	Fair
7. Hirschhorn et al. 2021 [30]	United States of America	Cross-sectional/ Emergency Medical Service providers/ 183		-	-	- Use of chemical cold packs. - Air-conditioning - Placing wet towels on affected person’s body. - Use of fluids for oral rehydration	-	Fair
8. Cahillane, 2014 [27]	United Kingdom	Policy document/ Residents of the State of New Hampshire		Excessive Heat Emergency Response Plan	- Elderly (65 years and above), infants and young children, pregnant women, the mentally ill, chronically ill persons, the homeless and poor, the socially isolated.	- Readiness of response teams and medical supplies to deploy. - Opening of cooling centres. - Orientation on guidelines to manage heat-related illnesses	- Increased public health awareness. - Information on staying hydrated. - Information to engage in protective actions e.g., wearing light clothing, eating light, easy to digest foods, taking frequent showers/baths, limiting outdoor activity.	Good
9. Errett 2023 [5]	United States	National Survey/ 38 United States jurisdictions		Provision of extreme heat public health preparedness plans and response activities	Persons with low incomes, elderly, the homeless, the chronically ill, outdoor workers	- Providing extended shelters for the homeless. - Surveillance of HRI cases. - Development of vulnerability heat map.	- Provision of communications on response activities. - Increase access to electric fans/ air-conditioning	Fair
10. Health Canada 2011 [29]	Canada	Policy document/ Residents of Canada		The Heat Alert Response System	Elderly, infants and young children, the chronically ill, physically-impaired, socially disadvantaged, tourists, workers, the physically active.	- Use of chemical cold packs. - Air-conditioning - Placing wet towels on affected person’s body. - Use of fluids for oral rehydration	- Provision of targeted groups with timely, consistent and accurate information on hydration, using cooling centres. - Community design to enable easy access to heat-relief places and drinking water. - Planting trees to decrease heat absorption and increase shade.	Good
11. UNICEF, Bangladesh 2024 [2]	Bangladesh	Policy document/ Residents of the Bangladesh		Provision of National guidelines on Heat-related illnesses. - Strengthening health system sustainability. - Promoting intersectoral collaboration	- Elderly (60 years and above), infants, children and adolescents, pregnant women, the mentally ill, chronically ill persons, the homeless and poor, the socially isolated.	- Readiness of response services and supplies to deploy during an emergency. - Wearing loose clothing. - Continuous orientation on guidelines to manage heat-related illnesses	- Tree planting. - Use of hollow blocks for buildings, solar reflective paint, double-glazed windows, roof-cooling materials for roofing, and ensuring cross ventilation. - Provision and dissemination of information on preventing HRIs e.g., staying hydrated, avoiding intense heat etc.	Good
12. Indian Institute of Public Health 2021 [10]	India	Regional policy document of Odisha / Residents of Odisha		- Setting-up of early warning and system preparedness to cope with heatwave - Provision and evaluation of town-specific heat action plans. - Provision of Health Advisory for prevention.	Women and children, infants and elderly, construction workers and traffic police, low-income persons and those with co-morbidities	- Identifying heatwave hotspots and vulnerable populations. - Putting Heat Action Plan that addresses heat-health risks. - Activating heat alerts.	- Provision of communications on response activities to build public awareness and ensure community outreach. - Advisory to reduce heat exposure. - Increase access to electric fans/ air-conditioning. - Capacity building among health care professionals	Good
13. WHO Europe, 2021 [15]	Europe	Policy document/ European countries		- Provision of National, regional and city heat guidance document. - Installation of heat health warning systems.	Patients aged 65 years and over with chronic circulatory and respiratory conditions	- Active surveillance of high-risk subjects during heat-waves. - Mobilization of at-risk patients to airconditioned rooms/ wards. - Indoor temperature measurements for all areas where patients reside and ensuring that cool areas are below 26°c. - Information dissemination to all concerned stakeholders on management protocols	- Information dissemination to all concerned stakeholders on preventing occurrence of HRIs, how to keep cool, staying hydrated etc. - Necessary retrofitting of building envelopes and Insulation. - Efficient active cooling. - Provision of external shading. - Ensuring adequate ventilation.	Good

## Discussion

This review was conducted with the aim of identifying health system strategies to tackle heat emergencies, members of populations targeted by these strategies, as well as emergency responses and interventions that have been used in ameliorating the harmful health effects of these emergencies in heat-prone areas. It was identified that certain members of the population apart from the elderly, were prone to these problems. Various countries were also able to enhance their healthcare delivery systems to more effectively deal with these problems through their locally-developed heat action plans (HAPs) and emergency protocols. In addition, various interventions were identified to further protect the public from developing these problems, if heat events were to occur.

This review identified various health system strategies across high-income and low/middle-income countries to enhance resilience against heat-related emergencies, and to provide optimal care. It reflected the action being taken by affected countries to safeguard their populace from the adverse impacts of extreme heat exposures, and also pointed-out that adopted strategies were specific for the differing areas. This does not however imply that their usefulness cannot be explored in other climes, as they can be useful pointers to areas requiring improvement in already established healthcare delivery systems [7, 13] Adopted strategies basically focused on ensuring that the necessary healthcare infrastructure was made available, alongside improving the capacity of healthcare workers, through regular training and retraining on managing HRIs. These are part of the vital components of the World Health Organization health system building blocks, which can also be applied in managing heat-related emergencies [7, 9, 16]. The availability and adequacy of medical supplies and medications should also be ensured [27] Another vital health system building block is the health information management system, which should be enhanced to provide real-time data on identified heat illnesses and daily mortalities. This is very useful in effective surveillance activities during heat-related events [5, 25, 26]. Other useful measures were also identified to include environmental modifications of landscape and building designs, to reduce the demands on healthcare resources during heat-related events. These offer lasting interventions that protects the populace from HRIs during extreme heat events and substantiates the need for effective inter-sectoral collaboration among government stakeholders in enhancing the heat-resilience of health systems [3, 24, 29]. In order to achieve this, as well as ensure effective healthcare leadership, the necessary health leadership structure should be put in place, which ensures that effective heat-resilient health systems can be established in these heat-prone areas [3]. 

Most persons reported to be targeted by the identified policies and strategies included elderly persons (65 years and above), infants, pregnant women, mentally ill persons, chronically ill persons who were on medications, the homeless and poor, as well as socially isolated persons [2, 10, 15, 22, 23, 25–27, 29]. Occupationally, construction workers, traffic police, among other outdoor workers were identified to be susceptible to these HRIs [10, 22, 25, 29]. Studies have shown that women who give birth during extreme heat periods are more likely to have neonates who develop illnesses, considering that heat stress can trigger foetal tachycardia, congenital defects, and other problems of foetal distress [29, 31]. These mothers are also at greater risks of developing pre-eclampsia and gestational hypertension, as well as gestational diabetes. Health care systems should thus target to keep these mothers as comfortable as possible and ensure that they are minimally exposed to heat stressors as much as possible [31]. It is also essential that in HAPs, alongside other groups of persons who are prone to developing HRIs, socially-disadvantaged persons should be identified and provided with effective care. This is being advocated as they may have less access to clean water, cool places, and health and social services [3, 10, 29]. The need for reduced exposure measures especially for occupationally-exposed persons who need to work outdoors, must also be considered in the development of these HAPs [5]. 

Together with building health system resilience against health emergencies, is the ability of the emergency response system to deploy the necessary emergency response mechanisms during heat events. It is vital that during heat-related emergencies, all vulnerable persons are prioritized during these response activities. This is necessary as they can be promptly taken care of and protected from progressing to more serious forms of HRIs [10, 26]. Heat warning systems were also reported to be very essential for an effective emergency response to be made possible [3, 7]. The widespread provision of cooling centres, chemical cold-packs, public education on heat-protective measures that can be adopted [23, 26, 30], as well as real-time surveillance, are also cardinal activities carried out in heat-prone areas [2, 5]. These timely emergency response activities are of paramount importance in preventing the development of severe heat-related illnesses like heat exhaustion and heatstroke, among exposed persons [32]. This is also hinged on the health information management system building block for enhancing the functionality of health systems [5, 25, 26]. Additionally, for these to be achieved, as well as ensure effective healthcare leadership, the necessary health leadership structure should be put in place, which ensures that effective heat-resilient health systems can be established in heat-prone areas [3]. There is also the need for regular assessment of these emergency protocols to ensure that they remain active and functional [3]. 

In the face of the worsening climatic outlook of the world, it is essential that just as health systems are made resilient to handle heat-related emergencies, the prevention of heat-related illnesses through public health interventions are enhanced [32]. This is necessary for minimizing the health, social, and economic burdens posed by extreme heat events in affected areas [32]. Public health interventions reported to be useful during heat-related events included the provision of free heat-health services [23], as well as building the capacity of the populace to handle heat-related events through heat-health awareness campaigns and community outreaches [5]. The provision of public orientation on preventive measures, including the need to stay hydrated, wear light clothes, taking frequent showers/baths, among others, have been shown to be very useful [2, 10, 15, 25–27]. This is due to its capacity to equip individuals with the needed knowledge and tools to protect themselves and others from the health risks associated with the occurrence of extreme heat [29]. Environmentally-designed interventions were also reported to be useful as effective direct solar emission interceptors. These included the use of trees, solar radiation interceptive building and paving materials (green roofs or high-α roofing/paving material [2, 24, 29]. Community designs that enabled easy access to cooling-centres and drinking water can also be beneficial [29]. 

This review had some limitations- the first being that quantifiable estimates of the effectiveness of health system strategies and public health interventions in mitigating the effects of heat-related emergencies were not provided. Apart from not being one of the review’s objectives, there is an apparent scantiness of research showing these estimates especially in low- and middle-income countries that experience heat-related emergencies. Despite the various actionable data from both low- and middle-income countries (LMICs) provided on the objectives of this review, it is essential that prospective research assessments are conducted to identify, by how much different public health interventions are able to protect the populace from the harmful effects of heat-related emergencies. In other words, what are the quantifiable estimates of these interventions on heat-related morbidities and mortality. Also, it is essential that simple, resource-friendly, tried and tested interventions that can be applied in LMICs for effective prevention of heat emergency health effects are identified and described in future research. It would also be interesting to identify the effect of uncontrollable factors (e.g. temperature) and controllable confounding factors (e.g. access to healthcare, and other social determinants of health), as well as other individual-level factors, on these interventions, using experimental research designs. In addition to the first identified limitation, the search strategy adopted was limited by language to only literature published in English language. This meant that reports in other languages were omitted. This was however done to avoid the problem of interpretation bias of literature published in non-English languages.

## Conclusion

This scoping review has been able to show the growing body of current research on heat emergency mitigation efforts by both global, regional and local healthcare stakeholders. It shows the need for the development of well-suited interventions that can be applied at region-specific levels based on the needs and resources available to them. There is the need for the regular assessment of heat action plans and intervention efforts to ensure that they meet the requirements of the populace they have been made for.

## Data Availability

No datasets were generated or analysed during the current study.

## Search strategy used in each database

**Table:**

s/*n*	Database	Search Strategy Used
1.	WHO Global Health Observatory	Heat, Heat stress
2.	PubMed	(health system response) OR (health system strategies)) OR (health system policies) AND (emergency preparedness) OR (emergency response) AND (heat-related illnesses) OR (heat-related health problems) OR (heatwave) OR (heatstroke) OR (extreme heat) OR (heat exhaustion) OR (heat syncope)
3.	Google Scholar	Targeted/vulnerable populations, health system strategies, emergency response, Public Health Interventions, “heat related illness”, - cold, - review.
